# Risk-adjusted observed minus expected cumulative sum (RA O-E CUSUM) chart for visualisation and monitoring of surgical outcomes

**DOI:** 10.1136/bmjqs-2024-017935

**Published:** 2024-11-25

**Authors:** Quentin Cordier, Hugo Prieur, Antoine Duclos, Top Surgeons Study Group, Jake Awtry

**Affiliations:** 1 Health Data Department, Hospices Civils de Lyon, Lyon, France; 2 Research on Healthcare Performance (RESHAPE), INSERM U1290, Université Claude Bernard Lyon 1 - Domaine de Rockefeller, Lyon, France; 3 Centre for Research in Epidemiology and Statistics (CRESS), METHODS Team, Université Paris Cité, Paris, France

**Keywords:** Statistical process control, Surgery, Healthcare quality improvement, Adverse events, epidemiology and detection

## Abstract

To improve patient safety, surgeons can continually monitor the surgical outcomes of their patients. To this end, they can use statistical process control tools, which primarily originated in the manufacturing industry and are now widely used in healthcare. These tools belong to a broad family, making it challenging to identify the most suitable methodology to monitor surgical outcomes. The selected tools must balance statistical rigour with surgeon usability, enabling both statistical interpretation of trends over time and comprehensibility for the surgeons, their primary users. On one hand, the observed minus expected (O-E) chart is a simple and intuitive tool that allows surgeons without statistical expertise to view and interpret their activity; however, it may not possess the sophisticated algorithms required to accurately identify important changes in surgical performance. On the other hand, a statistically robust tool like the cumulative sum (CUSUM) method can be helpful but may be too complex for surgeons to interpret and apply in practice without proper statistical training. To address this issue, we developed a new risk-adjusted (RA) O-E CUSUM chart that aims to provide a balanced solution, integrating the visualisation strengths of a user-friendly O-E chart with the statistical interpretation capabilities of a CUSUM chart. With the RA O-E CUSUM chart, surgeons can effectively monitor patients’ outcomes and identify sequences of statistically abnormal changes, indicating either deterioration or improvement in surgical outcomes. They can also quantify potentially preventable or avoidable adverse events during these sequences. Subsequently, surgical teams can try implementing changes to potentially improve their performance and enhance patient safety over time. This paper outlines the methodology for building the tool and provides a concrete example using real surgical data to demonstrate its application.

**WHAT IS ALREADY KNOWN ON THIS TOPIC**

- Monitoring surgical outcomes enables surgeons to assess changes in outcomes and identify areas for improvement.
- Choosing from available tools poses a dilemma between opting for intuitive yet simplistic visualisation tools and selecting more statistically rigorous approaches that may be overly complex for non-expert surgeons to interpret.

**WHAT THIS STUDY ADDS**

- We introduce a risk-adjusted observed minus expected cumulative sum (RA O-E CUSUM) chart, a novel visualisation tool inspired by statistical process control charts and designed for real-time monitoring of patient outcomes.
- This approach combines a user-friendly visual interface with the capability to detect statistically abnormal variations in surgical outcomes, signalling either deterioration or improvement in patient safety.

**HOW THIS STUDY MIGHT AFFECT RESEARCH, PRACTICE OR POLICY**

- The RA O-E CUSUM chart enables surgical teams to rigorously monitor outcomes and interpret variations over time, fostering behavioural change and facilitating the rapid implementation of solutions aimed at improving the reliability of surgical care.

## Introduction

 Serious and potentially preventable complications are common in surgery, affecting approximately 10% of patients.^1^ To enhance the quality of care, surgeons can employ statistical process control (SPC) tools to monitor surgical outcomes and their changes over time.^2^ Recognising that variability in production processes compromises quality, the control chart was initially introduced in the manufacturing industry by Walter Shewhart a century ago.^3^ This chart allows for timely evaluation to determine whether the monitored outcomes fall within the acceptable statistical limits, and if not it prompts investigations to uncover underlying causes and identify solutions to potentially improve the process. Since then, numerous tools have been proposed, including Shewhart’s original control chart for monitoring outcomes aggregated over consecutive periods, as well as Page’s cumulative sum (CUSUM) chart for providing real-time feedback.^4^ Subsequently, it was demonstrated that SPC tools could be adapted for use in the healthcare sector to detect changes in healthcare quality and safety,^5 6^ particularly in surgical settings.^7^ However, applying SPC from traditional industry to patient care is not straightforward.^8^ Unlike manufactured products, which are typically highly standardised, each surgical procedure and each patient is unique. Therefore, these tools must be adjusted to accommodate the heterogeneity in patient case-mix.^9 10^ Today, SPC is widely used with a variety of methodologies available, presenting a challenge for surgeons navigating the different tools.^11^

The selection process poses indead a significant dilemma. On one hand, intuitive and transparent options like the observed minus expected (O-E) chart are attractive but lack statistical rules in detecting significant variations in care safety.^12^,^14^ On the other hand, more sophisticated tools such as the CUSUM chart may provide greater rigour in data-driven interpretation, but their interpretation can be challenging for clinicians without corresponding statistical training.^14^,^16^ Our objective was to design an innovative tool by combining the O-E chart with the CUSUM chart, providing surgeons with a statistically relevant yet user-friendly solution for monitoring patient outcomes. We begin by outlining the definitions and frameworks of the O-E and CUSUM charts. Next, we introduce the combined risk-adjusted (RA) O-E CUSUM chart and demonstrate its application with real surgical data. Finally, we discuss its properties.

## Existing tools

Suppose an individual surgeon aims to monitor a binary surgical outcome, such as complications or mortality. The SPC family encompasses various control charts, each serving different purposes. For instance, the Shewhart p control chart aggregates surgical outcomes over consecutive periods (eg, months or quarters). However, this chart has slower detection speed because the results are only interpreted at the end of each period, provided that the sample sizes are sufficient. Alternatively, tools like the O-E, exponentially weighted moving average (EWMA), sequential probability ratio test (SPRT) and CUSUM charts operate on a procedure-by-procedure basis, allowing real-time monitoring of outcomes and facilitating prompt actions. Among these tools, the O-E chart is highly intuitive in visualising the safety of care over time but is relatively basic in its approach. In contrast, the CUSUM chart offers statistically rigorous interpretation and is highly sensitive in detecting shifts in outcomes,^17 18^ although it is considerably more complex to implement and interpret.

### O-E chart

The O-E chart allows the monitoring of binary outcomes by plotting the cumulative sum of the differences between observed and expected events (y-axis) across interventions (x-axis).^12 14^ The resulting curve on the O-E chart moves upward when an event ($y_{i}=1)$) occurs and moves downward in its absence ($y_{i}=0)$). It illustrates the cumulative deviation of the observed events from the expected count over interventions. When the probability of the event, $p_{0}$, is lower, the curve rises more sharply in case of an event, indicating a more significant deviation from the expected outcomes. Constructing an O-E chart is relatively straightforward:


(1) $$Unadjusted{\left(O-E\right)}_{n}=\;\sum_{i=1}^{n}\left(y_{i}-p_{0}\right)$$

**Figure 1:**
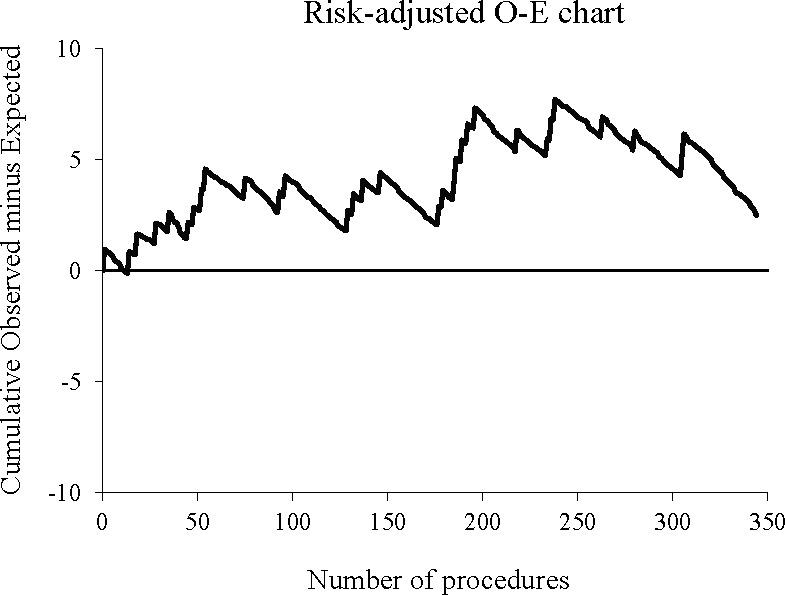
Example of a risk-adjusted observed minus expected (O-E) chart in thyroid surgery. The chart displays the occurrence of recurrent laryngeal nerve palsy after thyroidectomy for a single surgeon. The curve moves upward if the number of operations with recurrent laryngeal nerve palsy increases above that predicted by the risk model and moves downward if the number decreases. Data originated from a previous study: Duclos, A., Lifante, J.-C., Ducarroz, S., Soardo, P., Colin, C. and Peix, J.- L. (2011), Influence of Intraoperative Neuromonitoring on Surgeons’ Technique During Thyroidectomy. World J Surg, 35: 773-778 2014.https://doi.org/10.1007/soo268-011-0963-4.

The final value in the O-E represents the cumulative counts of potentially avoidable events if it is greater than 0, or potentially avoided events if it is lower than 0. To address the inherent risk variability among patients, an RA version of the O-E chart was introduced, also known as the variable life-adjusted display or the cumulative risk-adjusted mortality^12 19 20^ (see figure 1 for an example on thyroid surgery data^21^). This approach attributes to each intervention an expected probability of an event, denoted as $p_{i}$, which is typically derived from regression models identifying risk factors within the patient case-mix:


(2) $$Adjusted{\left(O-E\right)}_{n}=\sum_{i=1}^{n}(y_{i}-p_{i})$$

However, the traditional O-E chart does not allow the establishment of control limits, nor can it assess whether there is a statistically significant change in the rate of events. ‘Rocket-tail’ control limits, based on the percentiles of the marginal distribution of the cumulative sum, have been proposed^22^ but were shown not ideal in detecting changes in performance.^14^ Moreover, the presence of ‘good runs’ can mask ‘poor runs’ of interventions, misleadingly suggesting that the process is under control.^13^ Additionally, the O-E chart may not be the most appropriate tool to identify changes when sample sizes are small,^23^ a common scenario when monitoring outcomes at the individual surgeon level rather than at the institutional level.

### CUSUM chart

**Figure 2:**
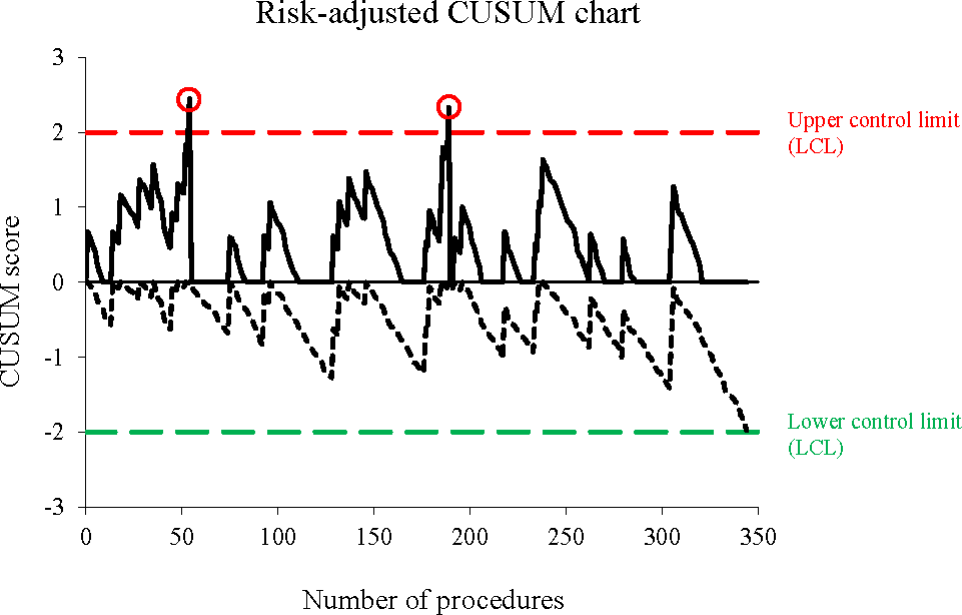
Example of a risk-adjusted cumulative sum (CUSUM) chart in thyroid surgery. The chart displays the occurrence of recurrent laryngeal nerve palsy after thyroidectomy for a single surgeon. The upper curve detects signals of deterioration in surgical performance, while the lower curve detects signals of improvement. When a curve crosses over its control limit, a signal is detected (shown with circles), indicating a statistically significant change in surgical performance. The graph is subsequently reset to allow further monitoring. In this example, deteriorations were detected at procedures 57 and 192. Data originated from a previous study: Duclos, A., Lifante, J.-C., Ducarroz, S., Soardo, P., Colin, C. and Peix, J.- L. (2011), Influence of Intraoperative Neuromonitoring on Surgeons’ Technique During Thyroidectomy. World J Surg, 35: 773-778 2014. https://doi.org/10.1007/s00268-011-0963-4.

Among all SPC tools, the CUSUM chart is frequently employed to monitor surgeons’ performance.^11^ In the context of a binary outcome, it determines whether the surgical process is under control (null hypothesis H_0_: event rate $\theta =\theta_{0}$) or out of control (alternate hypothesis H_1_: event rate $\theta =\theta_{1}$).^4^ This is achieved by calculating the cumulative sum of deviations between the observed outcome and a target value, and comparing it against predefined control limits. If the CUSUM score exceeds the control limit, the null hypothesis is rejected, indicating that the process is out of control. In its two-sided form, the CUSUM chart is decomposed into two subscores: one for detecting care deterioration trends ($X_{i}^{+}$) and another for detecting care improvement trends ($X_{i}^{-})$) (see figure 2). These subscores can be computed from log-likelihood ratio scores as follows:


(3) $$X_{i}^{+}=MAX\left(0;X_{i-1}+W_{i}\right)\;X_{i}^{-}=MIN\left(X_{i-1}-W_{i};0\right)\;\;\;with\;W_{i}=\left\{ \begin{array}{ll} \log\left(\frac{1-\theta_{1}}{1-\theta_{0}}\right) & \text{if }y_{i}=0\\ \log\left(\frac{\theta_{1}}{\theta_{0}}\right) & \text{if }y_{i}=1 \end{array}\right.$$

The control limits in CUSUM charts play a crucial role in determining the tool’s sensitivity and are associated with average run lengths (ARLs), which denote the average number of observations needed to detect a signal. Each specified control limit value, h, corresponds to an in-control ARL (ARL_0_), which we aim to maximise to avoid frequent false alarms, and an out-of-control ARL (ARL_1_), which we seek to minimise to detect signals promptly. Choosing appropriate control limits involves balancing between fast signal detection and minimising false alarms. These limits can be calculated directly, found in tables from the litterature or obtained through simulations based on in-control ($\theta_{0}$) and out-of-control ($\theta_{1}$) event rates^24^ (see online supplemental file 1).

An adjustment methodology proposed by Steiner *et al*^25^ addresses variations in preoperative risks among patients. This approach has already been applied numerous times.^26^,^28^ It includes a Markov chain-based approach for generating customised control limits. Here, R_0_ and R_1_ represent Odds Ratios (ORs) of events under the null and alternate hypotheses, respectively, where R_0_ is equal to 1 when the expected event probability, p_t_, is based on current conditions, and R_1_>R_0_ facilitates the detection of shifts. The RA CUSUM can be calculated as follows:


(4) $$X_{i}^{+}=MAX\left(0;X_{i-1}+W_{i}\right)\;X_{i}^{-}=MIN\left(X_{i-1}-W_{i};0\right)\;\;\;W_{i}=\left\{ \begin{array}{ll} \log\left(\frac{1-p_{i}+R_{0}p_{i}}{1-p_{i}+R_{1}p_{i}}\right) & \text{if }y_{i}=0\\ \log\left(\frac{\left(1-p_{i}+R_{0}p_{i}\right)R_{1}}{\left(1-p_{i}+R_{1}p_{i}\right)R_{0}}\right) & \text{if }y_{i}=1 \end{array}\right.$$

By employing control limits for signal detection, CUSUM charts allow for identification of special cause variations in care safety with statistical significance, whether indicating deterioration or improvement. However, CUSUM charts demand a deeper understanding of statistics and may present interpretation challenges for surgeons.^29^ In our study, we aimed to integrate CUSUM functionalities into the intuitive adjusted O-E chart, thereby enhancing accessibility and usability for surgical teams.

### The RA O-E CUSUM chart

To address the limitations of using the O-E chart and the CUSUM chart separately, several authors^14 30 31^ have recommended combining the two, with the CUSUM chart providing statistical backing for the O-E chart. Merging both tools into a single chart offers several key benefits. First, surgeons may only need to learn and use one tool. Second, using two tools simultaneously lacks simplicity,^16^ is more time-consuming, is prone to errors and is not ideal for dashboard integration. Lastly, it allows some statistical insights of the CUSUM chart to be displayed within the straightforward interpretive scale of the O-E chart. In this manuscript, we propose an RA O-E CUSUM chart aimed at efficiently monitoring binary surgical outcomes over time. Initially, the display of the RA O-E CUSUM chart is based on the RA O-E, illustrating the evolution of the difference between the observed events and the expected probabilities of events (refer to the O-E chart section for details). Subsequently, CUSUM-specific features are integrated into the chart to enable detection of significant variations in surgical safety.

The construction begins with a two-sided RA CUSUM chart designed to identify sequences of abnormal care safety. Users define their null and alternate hypotheses based on the shifts they wish to detect, and the corresponding CUSUM control limits are calculated accordingly. For each side, sequences are defined as the interval between the last procedure, with a CUSUM subscore at 0, and the first intervention, where the CUSUM subscore exceeds the control limit. These sequences are subsequently highlighted on the O-E chart, with red indicating deterioration and green indicating improvement. To maintain usability postdetection of abnormal variations, the CUSUM subscores are then reset to 0. Finally, the tool calculates the number of potentially avoidable events (in case of care deterioration) or the number of potentially avoided events (in case of care improvement) associated with each sequence by determining the difference in O-E values during that period. A step-by-step guide for constructing the tool is provided in online supplemental file 2 and a SAS code is provided in online supplemental file 3 (online at https://github.com/hugoprieur/OE-CUSUM). With this comprehensive tool, surgeons can easily visualise the evolution of surgical safety across procedures, identify periods of underperformance and overperformance and quantify the concrete impact on patient outcomes. Used in daily practice, they can investigate the causes of variations more promptly and potentially adjust their practices accordingly.

## Example based on surgical data

To illustrate the construction and implementation of the tool in a real surgical context, we retrospectively built the RA O-E CUSUM chart for a cohort of individual surgeons using data collected during a multicentre study in Lyon, France.

### Population and data set

Data were gathered from 14 surgical departments across four university hospitals in the Lyon area, France. We analysed surgical procedures performed by 45 surgeons specialising in cardiac, thoracic, digestive, endocrine, orthopaedic, urological and gynaecological surgery between 1 November 2020 and 31 December 2021. Exclusions applied to operations involving minors (under 18 years old), palliative care, organ donation or cases where patients declined to share their personal data (see online supplemental figure 1).

Prospective data collection used a uniform information system across Lyon university hospitals, encompassing sociodemographic details, care provided, surgical procedure specifics and primary diagnosis related to the surgical indication. Clinical research assistants supplemented this information with data sourced from patients’ electronic health records, detailing operations scheduling, intraoperative and postoperative adverse events, and preoperative comorbidities. Anaesthesia records contributed data on anaesthesia type, patients’ body mass index and the ASA (American Society of Anesthesiologists) physical status classification system. Table 1 provides an overview of the patients and the characteristics of the operations.

**Table 1:** Patients and operations characteristics

Characteristics	N=10 974
Male, n (%)	4822 (43.9)
Age, mean (SD)	56.4 (17.8)
Surgical specialty, n (%)	
Cardiac	962 (8.8)
Endocrine	824 (7.5)
Digestive	2576 (23.5)
Gynaecological	2208 (20.1)
Orthopaedic	3023 (27.5)
Thoracic	446 (4.1)
Urological	935 (8.5)
Scheduling of the operation (missing n=70), n (%)	
Elective	8844 (81.1)
Semi-urgent	348 (3.2)
Urgent	1712 (15.7)
Principal anaesthesia technique (missing n=11), n (%)	
General	8707 (79.4)
Regional	2025 (18.5)
Local	231 (2.1)
Initial surgical approach (missing n=75), n (%)	
Open	6818 (62.6)
Robot	492 (4.5)
Videoscopic	2474 (22.7)
Endoscopic	1115 (10.2)
ASA physical status classification system, n (%)	
1	3067 (27.9)
2	5042 (45.9)
3	2617 (23.8)
4	235 (2.1)
5	13 (0.1)
Median income in the city of residence in K€* (missing n=30), mean (SD)	23.0 (3.6)
Precarity, n (%)	938 (8.5)
Inpatient surgery (missing n=2), n (%)	8471 (77.2)
Comorbidities†, mean (SD)	2.4 (2.1)
HFRS frailty score of the patient during index stay, mean (SD)	1.1 (2.5)
Major adverse event included in the composite, including major complications potentially attributable to the surgeon (D0–D30 per postoperative procedure), n (%)	2109 (19.2)

Values are numbers (percentages) unless stated otherwise. Percentages might not sumtotal to 100 because ofdue to rounding.

* €1.00 (£0.86; US$1.08) as of May 2024.

† In order to take into account the preoperative risk of each operation, we have constructed a risk score based on all the variables presented in thisthe table as well as the following comorbidities: current pregnancy, obesity (BMIbody mass index ≥30 kg/m²), malnourishment, tobacco addiction, alcohol addiction, other addiction, open wound, surgical site infection, sepsis, endocarditis, cancer, neoadjuvant treatment, immune deficiency, coagulopathy, anticoagulant treatment, antiaggregation treatment, blood transfusion, coma, limb paralysis, other neurological disorder, confusion, dementia, depression, cardiovascular disease, neurovascular disease, peripheral arterial disease, cardiac arrhythmia, chronic heart failure, hypertension, diabetes, dyslipidemiadyslipidaemia, pulmonary artery systolic pressure (>60 mm Hg), chronic renal failure, acute renal failure, chronic respiratory failure, chronic obstructive pulmonary disease, liver disease, rheumatic pathology, and hypoparathyroidism.

ASA, American Society of Anesthesiologists; HFRS, Hospital Frailty Risk Score.

### Outcome

The outcome was a composite morbidity–mortality measure of major adverse events that occurred either during the surgical procedure or within the 30-day postsurgery period. It was inspired by the Clavien-Dindo classification^32^ and encompassed intraoperative or postoperative death regardless of the cause, postoperative transfer for at least two nights to intensive care unit or five nights to intermediate care unit due to organ failure, unplanned reoperation for complications related to the initial surgery and major surgical complications (see online supplemental file 4). The outcome scope covered a wide range of surgeon responsibilities, including aspects of perioperative care that might be influenced by external factors but remained under their supervision. A sensitivity analysis using an outcome more closely tied to the procedure, unplanned reoperations, is provided in online supplemental figure 2.

### Risk adjustment and the RA O-E CUSUM chart construction

To assess the surgical risk of each procedure, we designed a preoperative risk score using prediction models developed from a separate data set comprising patients who underwent surgeries performed by the same cohort of surgeons between 1 January 2022 and 31 October 2022. We constructed specific models for each of the seven surgical specialties through stepwise logistic regressions (entry threshold p=0.20, exit threshold p=0.10). We systematically considered a selection of variables, including patient age, gender, socioeconomic status, ASA score, an extensive list of mentioned comorbidities, surgical specialty, procedure complexity, indication, approach, scheduling and type of anaesthesia (see online supplemental file 4). Using the predicted risk for each surgical intervention, we separately constructed RA O-E and RA CUSUM charts for each of the 45 surgeons. For the CUSUM charts, control limits were determined via simulation of ARLs to detect deterioration if the adverse event rate doubled and improvement if the adverse event rate halved (refer to online supplemental file 1 for details). Subsequently, sequences indicating improvement and deterioration, along with the corresponding number of potentially avoidable or avoided events, were overlaid on the O-E chart to create the integrated RA O-E CUSUM chart. This combined chart allows visualisation of both O-E and CUSUM insights in a unified display. For a detailed step-by-step guide on constructing the RA O-E CUSUM chart, please refer to online supplemental file 2.

### O-E CUSUM chart interpretation

**Figure 3:**
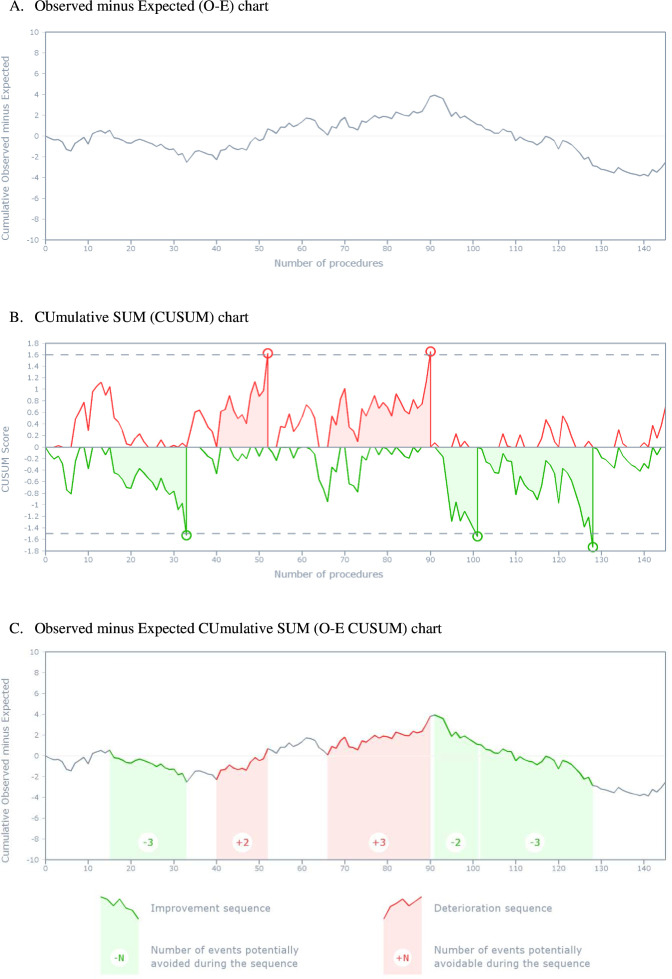
Risk-adjusted (A) O-E, (B) CUSUM and (C) O-E CUSUM charts for a single digestive surgeon (n=145 procedures performed between 1 November 2020 and 31 December 2021). The outcome was a composite morbidity–mortality measure of major adverse events occurring either during or in the 30 days following the surgery. (**A**) In the risk-adjusted O-E chart, the curve first moves downward since the number of major adverse events decreases below that predicted by the risk model, then slowly moves upward while the number of major adverse events increases, and finally moves downward again, with a final difference between observed and expected events of −2.5, that is, 2.5 events potentially avoided during the whole 14-month period. (**B**) In the risk-adjusted CUSUM chart, the coloured areas mark the sequences of deterioration and improvement, including all procedures between the signal and the last zero value of the subscore. The upper curve detects signals of deterioration in surgical performance at procedures 52 and 90, while the lower curve detects signals of improvement at procedures 33, 101 and 128. (**C**) In the risk-adjusted O-E CUSUM chart, sequences of deterioration and improvement are reported from the CUSUM chart to the O-E chart. First, the O-E CUSUM chart detects an improvement sequence (procedures 16–33, three events potentially avoided), followed by two distinct sequences of deterioration (procedures 41–52 and 67–90, respectively, two and three potentially avoidable events) and two consecutive sequences of improvement (procedures 92–101 and 102–128, respectively, two and three potentially avoided events).

RA O-E, CUSUM and O-E CUSUM charts generated for one of the 45 surgeons in our study are presented in figure 3. This surgeon, specialising in digestive surgery, conducted a total of 145 procedures between 1 November 2020 and 31 December 2021.

#### RA O-E chart

Figure 3A illustrates the RA O-E chart. The O-E chart initially shows that surgical performance exceeded expectations (the curve descends), then deteriorated until the 90th procedure before showing signs of improvement again. Overall, the difference between the observed and the expected events was −2.5, indicating that 2.5 events were potentially avoided during the period. However, this chart alone does not provide actionable insights beyond descriptive trends.

#### RA CUSUM chart

Figure 3B illustrates the RA CUSUM chart. The CUSUM chart identified five sequences of abnormal variations in care safety, where the safety of care significantly differed from the expected. Whenever the CUSUM score exceeded the control limit, whether indicating improvement (lower green curve) or deterioration (upper red curve), the tool detected a signal. Notable improvement sequences occurred during procedures 16–33, 92–101 and 102–128 (highlighted in green), while deterioration sequences were observed during procedures 41–52 and 67–90 (highlighted in red). However, this graph alone does not depict the practical consequences on patient outcomes.

#### RA O-E CUSUM chart

Figure 3C illustrates the RA O-E CUSUM chart. By overlaying the CUSUM on the O-E chart, the O-E CUSUM chart offers a comprehensive view of the surgeon’s performance. It reveals that the surgeon initially experienced a statistically significant improvement phase, avoiding three adverse events during procedures 16–33. This was followed by two distinct deterioration phases, where two and three adverse events were potentially avoidable beyond expectations during procedures 41–52 and 67–90, respectively. Subsequently, two consecutive improvement phases followed, with two and three potentially avoided events during procedures 92–101 and 102–128, respectively. This integrated view enables the surgeon to investigate the causes of these performance variations.

In this specific case, the start of the major improvement sequences coincided with the surgeon’s return to work after breaks: a 2-week hiatus between the 16th and 17th operations (during Christmas holidays) and a 4-week break between the 92nd and 93rd operations (summer holidays). The RA O-E CUSUM charts for all other surgeons are provided in online supplemental figure 3.

## Discussion

By integrating all the information from the O-E chart with the interpretable elements of the CUSUM chart, the RA O-E CUSUM chart provides users with both intuitive and statistically sound detections of improvement and deterioration sequences in patient safety. When a sequence of abnormal care safety is detected on the RA O-E CUSUM chart, the surgeon can immediately see the magnitude of the deviation from the expected outcomes. This visibility can be used to promptly consider actions that might address identified issues or capitalise on improvement trends. Overall, the RA O-E CUSUM chart enhances surgeons’ ability to monitor and interpret surgical performance comprehensively, facilitating proactive adjustments to practices based on real-time data analysis.

The majority of studies using SPC tools are based on either the O-E or the CUSUM chart.^11^ O-E charts provide intuitive visualisation by comparing observed outcomes against expected values, highlighting deviations that may indicate areas for improvement or concern in surgical safety. On the other hand, CUSUM charts offer statistical rigour in detecting shifts from expected outcomes over time, distinguishing between improvement and deterioration phases with defined control limits. There is strong advocacy to integrate these two tools together, combining their respective strengths.^16^ Several authors have suggested simply overlaying CUSUM chart signals onto the O-E chart.^30 31^ Sherlaw-Johnson^33^ also proposed adding control limits to the O-E chart, based on an underlying CUSUM. These control limits represented the thresholds the O-E curve needed to reach for the CUSUM chart to detect a signal. Sun and Kalbfleisch^34^ introduced a time-continuous adaptation of the O-E chart, featuring CUSUM-like control bands inspired by Sherlaw-Johnson’s^33^ work. Yue *et al*^35^ proposed an O-E EWMA chart similar to Sherlaw-Johnson’s but with the control bands based on an EWMA chart instead of a CUSUM chart. Finally, Wittenberg *et al* incorporated V-masks into the O-E chart.^16^ V-masks are V-shaped cursors applied to the O-E curve at each observation, signalling when one of the V’s arms intersects the curve.^36^ Signal detection with the V-mask is equivalent to detection by the CUSUM, as shown by Sun and Kalbfleisch.^34^ Other SPC tools, such as SPRT charts derived from the CUSUM principles, offer a score based on log-likelihood ratios but do not differentiate between deterioration and improvement processes.^37^ EWMA charts provide a weighted approach to recent observations, smoothing out fluctuations, but may introduce inertia in detecting significant changes, limiting their utility in real-time surgical settings.^38^ Incorporating features from these various SPC tools into a unified approach, possibly within an O-E CUSUM framework, could enhance the capability to monitor surgical outcomes comprehensively. This integrated approach would leverage the strengths of each tool—intuitive visualisation, statistical sensitivity and real-time responsiveness—to provide surgeons with actionable insights for enhancing patient safety and quality of care.

The proposed RA O-E CUSUM chart offers a distinctive approach by representing the difference between observed and expected values on the O-E chart rather than the raw CUSUM score. This metric provides surgeons with a tangible measure to assess the cumulative impact of changes in surgical outcomes over time. Instead of overlaying control limits or visual layers, the proposed RA O-E CUSUM chart displays sequences of change in surgical performance. This ensures the user’s attention is drawn to the entire statistically significant sequence, from start to end, preventing surgeons from fixating on isolated procedures (see online supplemental figure 4). Unlike focusing solely on the CUSUM score, which remains sensitive to large deviations between observed and expected values, our approach requires multiple consecutive deviations to trigger a signal, ensuring that changes are displayed only when they are statistically relevant. The RA O-E CUSUM chart effectively smooths out short-term variations, allowing surgeons to focus on longer-term trends in surgical performance. This strategic view is crucial for implementing actions aimed at improving patient safety consistently over time. The chart can handle multiple signals and enables surgeons to quantify the magnitude of statistically significant changes in terms of numbers of avoidable or avoided events. By using a colour-coded system (red for deterioration, green for improvement), this chart also simplifies the identification of trends in patient safety. This results in an easy-to-read tool that does not require an understanding of how the CUSUM chart works or the concept of control limits, making its interpretation accessible to surgeons, even without statistical background.

Some limitations persist in introducing our tool. While the RA O-E CUSUM chart is easier to interpret than the traditional CUSUM chart, it retains the same underlying statistical foundations. These include initial assumptions such as thresholds for identifying when the process is out of control, statistical adjustments to manage preoperative risks specific to each procedure and the calculation of CUSUM scores and control limits, all of which are crucial to the tool’s implementation. Furthermore, although daily use does not require specific skills, the tool’s overall design complexity remains a consideration. Lastly, the inability to display the CUSUM score or indicate how fast control limits are exceeded means that users cannot visualise the inertia leading to signal detection. This lack of transparency may lead users to perceive the tool as a black box. In summary, while the RA O-E CUSUM chart offers improved interpretability, addressing these complexities and enhancing transparency are essential to maximise user understanding and trust in its outcomes.

## Conclusion

By providing an intuitive and statistically sound tool capable of real-time monitoring of surgical outcomes and detection of abnormal changes in patient safety, we empower surgeons to promptly investigate potential causes of these variations. This might enable them to take swift action and try to mitigate risks in cases of statistically significant deterioration, or conversely reinforcing effective practices during periods of improved outcomes.

## Supplementary material

10.1136/bmjqs-2024-017935 online supplemental file 1
