# Supplementary material for: Risk-adjusted observed minus expected cumulative sum (RA O-E CUSUM) chart for visualisation and monitoring of surgical outcomes
Source: BMJ Qual Saf. 2024 Nov 25;34(5):e017935. doi: 10.1136/bmjqs-2024-017935 (PMC12013565; doi:10.1136/bmjqs-2024-017935)
Supplement: online supplemental file 1 [file bmjqs-34-5-s001.pdf]

Supplemental File 1 : CUSUM chart control limits

Control limits are essential for the detection of signals of abnormal variation in the safety of care. The CUSUM chart is based on a hypothesis test:

- H0 : the event rate has not changed, the statistical process is in-control
- H1 : the event rate has changed, the statistical process is out-of-control

To identify whether the adverse event rate of the process has changed, we first need to quantify the changes we aim to detect. In our case, we considered as deteriorations processes where the event rate doubled and as improvements processes where the event rate halved. We will then test these hypotheses after each surgical procedure to determine whether the process is in control or out of control, whether for deterioration (X_t^+ subscore) or improvement (X_t^- subscore). In practice, this test involves comparing each subscore with control limits that will reflect the minimum detectable change. Note: contrary to common belief, the lower control limit is not necessarily the symmetrical counterpart of the upper control limit! If the subscore value is beyond the control limit, the null hypothesis will be rejected, and the process will be considered as out-of-control.

- No rejection of H0 if $X_t^+ \leq h^+$ et $X_t^- \geq h^-$ ➔ in-control process
- Rejection of H0 if $X_t^+ > h^+$ et $X_t^- < h^-$ ➔ out-of-control process

How can be determined the control limits h^+ and h^-, so that they reflect the changes we want to measure? Detecting a signal does not mean with certainty that the process is out-of-control, but rather that there is enough evidence to think that the process is indeed out-of-control. This lack of certainty highlights that the risk of error exists and that the CUSUM may detect false positive signals. Therefore, control limits must be chosen to be large enough to reduce this risk, without eliminating the chances of detecting true positives. The ideal control limit choice is based on the average run lengths (ARL) of the CUSUM under H0 (ARL0) and H1 (ARL1):

- ARL0 is the average number of procedures needed before detecting a signal when the process is in control. This represents the average time before detecting a false positive, a risk we want to avoid as much as possible. Ideally, ARL0 should be as large as possible to delay false positive detection as long as possible.
- ARL1 is the average number of procedures needed before detecting a signal when the process is out of control. This represents the average time before detecting a true positive signal, which we want to detect as quickly as possible. Ideally, ARL1 should be the smallest possible.

Each control limit value corresponds to an ARL0 and an ARL1. The aim is then to find a compromise by setting h so that ARL0 is as large as possible and ARL1 is as small as possible. Several methods are possible to achieve this:

Determination of h from ARL tables:

ARL tables are available in the literature and provide ARL0 and ARL1 values, given in-control and out-of-control event rates and each value of h. A quick look on the tables is usually enough to find a good compromise. These tables are however only applicable to non-adjusted CUSUM charts.

Determination of h by simulation (approach used in our example):

The idea is to simulate a large number of processes (at least 1000) both in-control and out-of-control, and calculate for different values of h the number of procedures in average before the in-control processes detect signals (ARL0, false positive) and conversely the number of procedures in average before out-of-control processes detect signals (ARL1, true positive). This can be done by resampling (bootstrap approach) preoperative risks obtained from the data used to construct the adjustment models (i.e. models fitted values):

- Simply resampling will enable to generate new in-control processes.
- Resampling with changes in the event rate will enable to generate out-of-control processes. In the case of adjusted CUSUM charts, it is important to verify that after odds ratios of out-of-control processes (compared to in-control processes) match the initial out-of-control hypotheses. In our case, we resampled to generate processes with a doubling (h^+) and a halving (h^-) in the adverse event rate (see figure and table below):

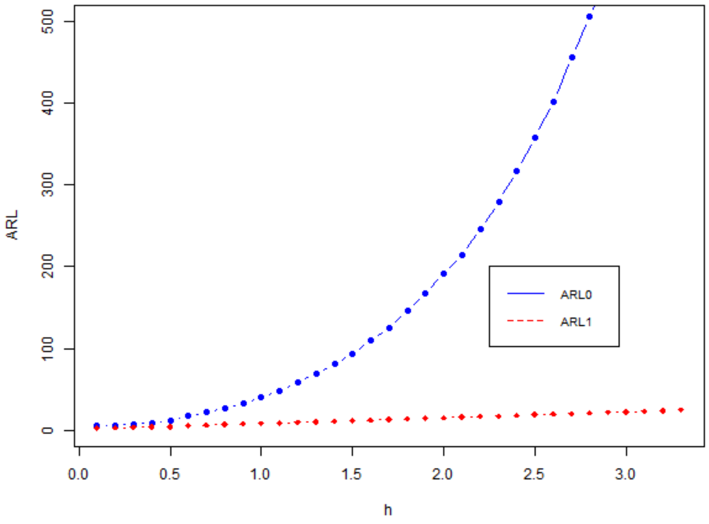

ARL0 and ARL1 for different values of h^+

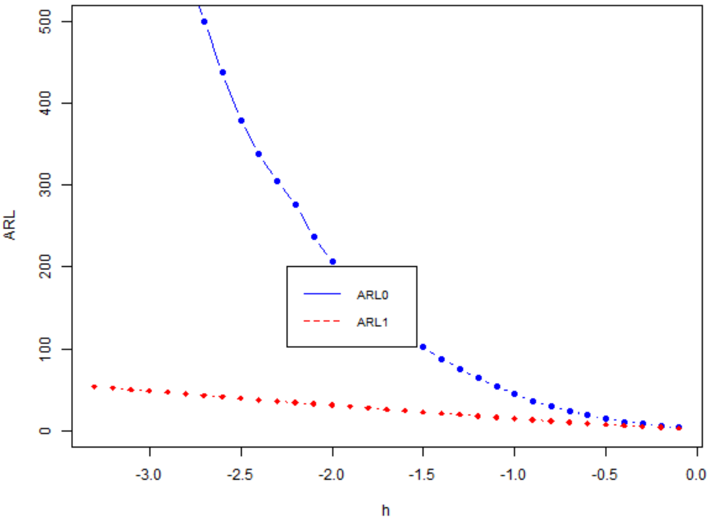

ARL0 and ARL1 for different values of h^-

As shown in these charts, the larger the absolute values of h, the higher the ARLs, with ARL0 increasing faster than ARL1. Thus, the goal is to set the value of h that maximizes ARL0 while minimizing ARL1. In our example, we arbitrarily selected the smallest values of h that ensured ARL0 > 100. The corresponding ARL1 values were relatively low, providing satisfactory sensitivity. However, this decision should be made on a case-by-case basis, depending on the specific situation and the level of sensitivity the user wishes to assign to the chart.

Corresponding ARL0 and ARL1 for different values of h^{+} aand h^{-} :

Upper control limit h^{+}			Lower control limit h^{-}		
h+	ARL0	ARL1	h-	ARL0	ARL1
0.1	5.255	3.074	-3.3	995.738	53.666
0.2	5.976	3.233	-3.2	887.802	51.96
0.3	7.189	3.506	-3.1	812.609	50.163
0.4	8.781	3.881	-3	744.276	48.498
0.5	12.14	4.486	-2.9	650.041	46.496
0.6	17.051	5.343	-2.8	590.469	44.781
0.7	21.302	6.272	-2.7	517.782	43.156
0.8	25.446	6.679	-2.6	459.851	41.565
0.9	31.52	7.23	-2.5	397.479	40.192
1	39.151	7.888	-2.4	360.911	38.499
1.1	47.313	8.608	-2.3	319.571	37.072
1.2	56.573	9.395	-2.2	282.343	35.359
1.3	66.048	10.182	-2.1	251.719	33.726
1.4	76.233	10.741	-2	215.841	31.824
1.5	87.879	11.424	-1.9	191.95	29.927
1.6	101.73	12.09	-1.8	165.273	28.258
1.7	117.128	12.867	-1.7	141.81	26.636
1.8	138.447	13.528	-1.6	122.886	25.069
1.9	158.988	14.348	-1.5	106.582	23.303
2	186.429	15.017	-1.4	90.097	21.612
2.1	215.675	15.621	-1.3	78.84	19.955
2.2	258.823	16.419	-1.2	66.644	18.34
2.3	284.911	17.014	-1.1	55.763	17.047
2.4	333.289	17.745	-1	46.116	15.488
2.5	378.305	18.369	-0.9	37.652	13.937
2.6	421.509	19.072	-0.8	29.958	12.436
2.7	474.041	19.815	-0.7	24.483	10.874
2.8	522.141	20.502	-0.6	18.655	9.213
2.9	582.672	21.182	-0.5	14.453	7.895
3	631.362	21.915	-0.4	10.633	6.526
3.1	714.66	22.505	-0.3	8.05	5.272
3.2	797.41	23.184	-0.2	5.584	4.261
3.3	905.746	23.877	-0.1	3.573	3.079

We have hence opted for the following values:

- h^{+} = 1.6, ARL0 = 101.73 and ARL1=12.09
- h^{-} =-1.5, ARL0 = 106.58 and ARL1=23.30

Determination of h by Markov chain methodology

Steiner et al. (Steiner SH, Cook RJ, Farewell VT, Treasure T. Monitoring surgical performance using risk-adjusted cumulative sum charts. Biostat Oxf Engl. 2000 Dec;1(4):441–52.) propose calculating control limits using a Markov chain approach. Markov chains are random processes that transition through different states (e.g., good health, illness, death), with state changes represented by a transition matrix. In the case of a CUSUM process, the states represented in the matrix include all possible values that the process can take over time. Each value in the matrix represents the probability of transitioning from one state to another. After some calculations, it is possible to calculate the matrix coefficients and derive an equation that connects average run lengths (ARLs) to control limits.

Supplemental File 2 : Step-by-step guide to build the Risk-adjusted Observed minus Expected Cumulative Sum (O-E CUSUM) chart

Step 0: risk-adjustment

The preliminary step in the O-E CUSUM construction process is to determine whether or not the tool requires adjustment. Usually, adjustment is essential as it allows for consideration of the specific preoperative risk associated with each surgical procedure. Generally, this is done by fitting regression models on a different dataset and applying these models to the data to be represented on the tools. It is then possible for each surgical procedure to generate predicted values that will correspond to the preoperative risk. Statistical adjustment can be complex, and its quality can have significant consequences on the interpretability of the tool:

- In case of an underlying secular trend, characterizing something happening at the macroscopic scale (e.g. a continuous deterioration related to patients being increasingly severe, and consequently a case-mix that is not fully captured by the variables), it is necessary to account for it to avoid systematically detecting improvements or deteriorations in surgical performance. Indeed, the tool is intended to detect abnormal variations in the safety of care.
- If the models are unstable, poorly calibrated, or not predictive, there is a high risk of calculating biased preoperative risks, making the interpretation of the tools unreliable.

It is also possible to consider using hierarchical regression models, such as random effects models or generalized estimating equation (GEE) approaches, to account for the case-mix of surgical procedures specific to each surgeon. However, this requires having sufficient data for each surgeon in the dataset used to design the models. In our example, we have preferred to opt for logistic regression models, so that we could provide the tool to new surgeons without necessarily having prior data about them.

Step 1 : choosing hypotheses

The construction of the O-E CUSUM requires setting hypotheses to configure the CUSUM and define the detection sensitivity of the tool :

- Null hypothesis H_0: the process is in-control
- Alternate hypothesis H_1: the process is out-of-control, either improving or deteriorating

For an unadjusted tool, this means setting the expected rate of event when the process is in-control (θ_0) and when the process is out-of-control (θ_1). Since the safety of care can either improve or deteriorate, we will consider two different values for θ_1, namely θ_1^- and θ_1^+. A signal will be detected for deterioration if $\theta > \theta_1^+$ and for improvement if $\theta < \theta_1^-$.

For an adjusted tool, it is no longer θ_0 and θ_1 that we need to set but rather R_0 and R_1, which are respectively the odds ratios under the null and the alternate hypothesis. When the surgical risk is based on current conditions, we generally set $R_0 = 1$. In our example, we chose to set $R_1^- = 0.5$ (signal for improvement if the risk of adverse event halved) and $R_1^+ = 2$ (signal for deterioration if the risk doubled).

Step 2: construction of the Observed minus Expected (O-E) chart

First, surgical procedures performed by the surgeon must be sort in chronological order. For each procedure, we will then calculate the difference between observed outcomes ($y_i = 1$ if an event occured et $y_i = 0$ if no event occured) and the expected risk (p_0 or p_t, depending on whether the tool was adjusted or not). This difference is positive if $y_i = 1$ and negative if $y_i = 0$. The absolute value of this difference increases as the gap between the predicted and the observed value increases. Then, we will calculcate the cumulative sum of the differences, procedure by procedure. Finally, we will plot the evolution of the cumulative sum in function of the number of the procedure (it is also possible to use a temporal scale as x-axis). The curve will rise if more events than expected occur, stagnate if the number of observed events matches the number of expected events, and fall if fewer events are observed than expected. The final value of the O-E chart represents the number of potentially avoidable (if positive) / avoided (if negative) events over the entire period.

Step 3: construction of the CUmulated SUM (CUSUM) chart

The CUSUM chart enables the detection of sequences of improvement and deterioration. Even if the chart is not strictly visible in the O-E CUSUM chart, its construction remains essential. This can be done in two steps: first by calculating the control limits (see supplemental file 1), and then by calculating the CUSUM subscores.

The surgeon's surgical procedures must again be sorted chronologically. Then, for each surgical procedure, we will calculate the weights based on the log-likelihood ratio scores (W_t, see equations (3) and in the manuscript (4)). From these weights, we can derived the CUSUM subscores are derived: X_t^+ (detection of sequences of deterioration) and X_t^- (detection of sequences of improvement). After each procedure, a hypothesis test is performed and the subscores are compared to the control limits. If the control limits are not reached ($X_t^+ < h^+$ and $X_t^- > h^-$), the null hypothesis is not rejected and the process is considered in-control: monitoring can continue as usual. However, should the control limits be reached, the null hypothesis would be rejected, with detection of a signal. The process can then be considered out-of-control, indicating an abnormal variation in the safety of care. This variation would be considered as a deterioration if involving the X_t^+ subscore concerned, and as an improvement if the involved subscore is X_t^-. The sequence of abnormal variation must be then identified as all surgical procedures between the last subscore value equaling 0 and the value where the signal was detected. The subscore is then reset to 0 to pursue monitoring.

Finally, subscores are plotted separately according to the number of procedure. Sequences of abnormal variations in patient safety must then be reported, using colored areas (red for deterioration sequences in the upper chart and green for improvement sequences in the lower chart). These sequences we will transcripted to the O-E CUSUM chart.

Step 4: construction of the O-E CUSUM chart

The final step in the construction is to assemble the O-E CUSUM chart. First, we will display the sequences of abnormal variations detected by the CUSUM chart to the O-E chart. Then, we will for each detected sequence then sum the differences between observed and expected values,

from the first to the last surgical procedure in the sequence. This value will correspond to the number of avoidable events (positive value, deterioration sequence) / avoided events (negative value, improvement sequence) and will be added to the chart (we opted for bubbles). The tool is now ready for use!

Supplemental File 3 : SAS code to generate the RA O-E CUSUM chart

```
/*



O-E CUSUM GRAPH SAS Code v1.0, 11/10/2024
This SAS code can also be found online at https://github.com/hugoprieur/OE-CUSUM

Draws the O-E CUSUM graph of a given dataset of procedures into a png file.



Inputs :
> CUSUM Parameters
> Table of procedures including the following variables :
       - Number of the procedure in the dataset
       - Expected probability of event
       - Observed event

Output :
> png file of a O-E CUSUM graph



Summary :
> Parameters
       CUSUM parameters for the detection of signals and output parameters,
including path, name and size of the output file
> Sample data
       A table of simulated data is provided for testing the code.
> O-E, CUSUM scores and signals
       Calculation of the observed minus expected values, CUSUM scores, and CUSUM
signals of improvement or deterioration
> Annomac tables
       Creation of datasets for the different graphic elements that will be drawn
by the annotate procedure
> Graph file output



*/







/* -------------------------------- */
/* ---------- Parameters ---------- */
/* -------------------------------- */



/* --- CUSUM parameters --- */

%LET R0 = 1;
%LET RA_X = 2;             /* RA for deterioration signals */
%LET RA_Z = 1/2;     /* RA for improvement signals */
%LET LIM_X = 1.6;    /* CUSUM limit for deterioration signals */
%LET LIM_Z = -1.5;   /* CUSUM limit for improvement signals */



/* --- Output --- */

%LET PNG_PATH = C:\folder\O-E_CUSUM.png; /* Path to the png file to create */
```

```
%LET XPX = 1920;           /* Width of the picture (graph + labels) (pixels) */
%LET YPX = 680;            /* Height of the picture (graph + labels) (pixels) */
%LET LINESIZE = 0.3;       /* Lines base width */
%LET FONTSIZE = 3;         /* Size of the tick labels */
%LET X_INTERVAL = 10;      /* Interval between 2 ticks on the x-axis */
%LET Y_INTERVAL = 2;       /* Interval between 2 ticks on the y-axis */
/* Colors (format : "CX" & hexadecimal) */
%LET CX_AXIS = CX8B9AAB;
%LET CX_STD         = CX8B9AAB;
%LET CX_SEQX = CXFF3030;
%LET CX_SEQZ = CX14B700;
%LET CX_SEQX_L      = CXFFEAEA;
%LET CX_SEQZ_L      = CXEAFFEA;
%LET CX_AXIS_L      = CXF5F5F5;




/* ---------------------------------- */
/* ---------- Sample data ---------- */
/* ---------------------------------- */




data base;
      input n pred evt;
      datalines;
1 0.218324376 0
2 0.000756346 0
3 0.000003651 0
4 0.004897523 0
5 0.000259069 0
6 0.271548888 0
7 0.168342343 1
8 0.000553514 0
9 0.022767651 0
10 0.00001497 0
11 0.65536515 1
12 0.38359939 1
13 0.52092919 1
14 0.96276556 1
15 0.00333328 0
16 0.99551418 1
17 0.00000065 0
18 0.56107034 0
19 0.29265058 1
20 0.00051051 0
21 0.00000098 0
22 0.96527270 1
23 0.01761614 0
24 0.64638632 1
25 0.23484585 0
26 0.96729063 1
27 0.94647897 1
28 0.00000327 0
29 0.00001233 0
30 0.55219308 1
31 0.85178232 1
32 0.90062453 1
33 0.18860821 0
34 0.02508199 0
35 0.14915877 0
36 0.00353451 0
37 0.32154201 0
38 0.00114298 0
```

```
39 0.00005086 0
40 0.00000534 0
41 0.00000234 0
42 0.99025254 1
43 0.07440102 0
44 0.98671174 1
45 0.74435805 1
46 0.26070163 0
47 0.00004238 0
48 0.92010631 1
49 0.83979927 1
50 0.00026351 0
51 0.00106049 0
52 0.23718294 0
53 0.56763473 1
54 0.01243145 0
55 0.01104642 0
56 0.00000163 0
57 0.58087298 0
58 0.11157097 1
59 0.99433715 1
60 0.63178705 0
61 0.40258098 0
62 0.07778404 0
63 0.27430608 0
64 0.04810524 0
65 0.00018669 0
66 0.15107899 0
67 0.54245928 1
68 0.84487540 1
69 0.37220112 1
70 0.14293314 0
71 0.05222025 0
72 0.22778525 0
73 0.02712744 0
74 0.12785133 0
75 0.88084493 0
76 0.05020076 0
77 0.06987377 0
78 0.02454716 0
79 0.98359869 1
80 0.00761436 0
81 0.97979134 1
82 0.29090464 0
83 0.00819029 0
84 0.00002407 0
85 0.00963778 0
86 0.00009441 0
87 0.19495348 0
88 0.33855396 0
89 0.01346008 0
90 0.00000801 0
91 0.00069185 0
92 0.00000119 0
93 0.52311713 1
94 0.00348021 0
95 0.80765306 0
96 0.00002071 0
97 0.03329127 0
98 0.04994371 0
99 0.17862950 0
100 0.8395834 1
101 0.6986946 1
102 0.0000006 0
103 0.0001226 0
104 0.3073143 0
105 0.1713220 0
106 0.9300953 1
```

```
107 0.9444526 1
108 0.0000001 0
109 0.0002982 0
110 0.2309132 0
111 0.0000708 0
112 0.2516242 0
113 0.0000005 0
114 0.9905170 1
115 0.0780732 0
116 0.0014393 0
117 0.9938476 1
118 0.0093629 0
119 0.8780204 1
120 0.9741164 1
121 0.0000922 0
122 0.0000808 0
123 0.9962916 1
124 0.0066511 0
125 0.0832514 0
126 0.0679356 0
127 0.0000044 0
128 0.4551175 1
129 0.1738783 0
130 0.0000248 0
131 0.0074039 0
132 0.0519059 0
133 0.0145781 0
134 0.9752933 1
135 0.0000024 0
136 0.6220216 1
137 0.8291508 1
138 0.0000173 0
139 0.0001830 0
140 0.0000020 0
141 0.7536943 1
142 0.0000007 0
143 0.9406913 1
144 0.0017504 0
145 0.7094722 1
146 0.0001033 0
147 0.4541259 1
148 0.2601301 0
149 0.0002826 0
150 0.5896005 1
;
run;




/* ---------------------------------------------------- */
/* ---------- O-E, CUSUM scores and signals ---------- */
/* ---------------------------------------------------- */



/* --- Calculating observed minus expected, CUSUM scores and CUSUM signals */

data cc (keep = n evt pred x z oe signal);
      set base (where = (evt ne . and pred ne .));
      retain x 0 z 0 oe 0; /* x : CUSUM deterioration score, z : CUSUM 
improvement score, oe : observed minus expected */
      signal = " "; /* "x" if the procedure triggers a deterioration signal, "z" 
if it triggers an improvement signal, else empty */
      /* Resetting both CUSUM scores after a signal (sequences cannot overlap) */
      if x > &LIM_X. or z < &LIM_Z. then do;
            x = 0;
```

```
                z = 0;
        end;
        /* Calculating the observed minus expected */
        oe = oe + evt - pred;
        /* Calculating both CUSUM scores in the case of an event */
        if evt = 1 then do;
                w_x1 = log( (1 - pred + &R0 * pred) * &RA_X / (1 - pred + &RA_X *
pred) / &R0 );
                w_z1 = log( (1 - pred + &R0 * pred) * &RA_Z / (1 - pred + &RA_Z *
pred) / &R0 );
                x = max(0, x + w_x1);
                z = min(0, z - w_z1);
        end;
        /* Calculating both CUSUM scores in the case of no event */
        if evt = 0 then do;
                w_x0 = log( (1 - pred + &R0 * pred) / (1 - pred + &RA_X * pred) );
                w_z0 = log( (1 - pred + &R0 * pred) / (1 - pred + &RA_Z * pred) );
                x = max(0, x + w_x0);
                z = min(0, z - w_z0);
        end;
        /* CUSUM signal triggers */
        if x > &LIM_X. then signal = "x";
        if z < &LIM_Z. then signal = "z";
run;



/* --- Identifying CUSUM sequences : groups of consecutive procedures that lead
to a signal --- */
/* Sequences start at the last procedure whose CUSUM score was 0, and end at the
procedure that signalled */
/* All procedures are divided into groups : each sequence is a group, then all
procedures between two given sequences form groups */

proc sort data = cc; /* The dataset has to be sorted in reverse for the next data
step */
        by descending n;
run;
data cc;
        set cc;
        retain group 1 group_type "stand"; /* group : id number of the group ;
group_type = "stand" (no sequence), "seq_x" (deterioration sequence) or "seq_z"
(improvement sequence) */
        /* If we reach the start of a sequence, we start a new group ; it is
assumed not to be a sequence, but this might change on the last 2 "if" blocks */
        /* Note that we are reading the dataset in reverse order, so the first
procedure of a sequence is the last one we read */
        if x = 0 and group_type = "seq_x" then do;
                group = group + 1;
                group_type = "stand";
        end;
        if z = 0 and group_type = "seq_z" then do;
                group = group + 1;
                group_type = "stand";
        end;
        /* End of a sequence -> new group (note that this is the first procedure we
read from this sequence) */
        if signal = "x" then do;
                group = group + 1;
                group_type = "seq_x";
        end;
        if signal = "z" then do;
                group = group + 1;
                group_type = "seq_z";
        end;
run;
proc sort data = cc; /* Going back to chronological order */
        by n;
```

```
run;



/* --- Calculating the observed minus expected value within a sequence, and
storing the procedure the sequence starts at --- */

data cc (drop = lag_n);
       set cc;
       retain group_oe . n_seq_start . lag_n .; /* group_oe : progression of the
O-E during the sequence, n_seq_start : first procedure of the sequence, lag_n :
previous procedure */
       /* Start of a sequence */
       if group_type in ("seq_x" "seq_z") and group ne lag(group) then do;
              group_oe = oe - (evt - pred); /* Storing the last O-E value before
the sequence */
              n_seq_start = lag_n; /* Id of the previous procedure */
              if _n_ = 1 then do; /* In case of a sequence starting at the first
procedure */
                     group_oe = 0;
                     n_seq_start = 0;
              end;
       end;
       /* End of a sequence */
       if signal ne " " then group_oe = int(oe - group_oe); /* group_oe was set at
the O-E value at the start of the sequence */
       /* No sequence -> resetting the values */
       if group_type = "stand" then do;
              group_oe = .;
              n_seq_start = .;
       end;
       /* Updating lag_n */
       lag_n = n;
run;





/* -------------------------------------- */
/* ---------- Annomac tables ---------- */
/* -------------------------------------- */



/* --- Prerequisites --- */

%annomac;

/* Format for the O-E values within sequences */
proc format;
       picture signe
       low - < 0 = '000'(prefix = '-')
       0 = '0'
       0 < - high = '000' (prefix = '+');
run;



/* --- Axis --- */

/* Calculating the x-axis end value */
data _null_;
       set cc;
       call symput("N_MAX", _n_);
run;

/* Calculating the y-axis end values */
```

```
data _null_;
	set cc;
	retain oe_max 0;
	if oe >=0 then oe_max = max(oe_max, oe * 1.5); /* We add some room on top
of the curve... */
	if oe < 0 then oe_max = max(oe_max, oe *-2.5); /* ... and more room below
it (accounting for the O-E value circles) */
	call symput("OE_MAX", put(round(oe_max, &Y_INTERVAL.), 3.)); /* Rounding to
the nearest interval */
run;

/* Using annomac macros */
data axis;
	format color $8. function $8. style $12. text $39. position $1.;
	retain hsys xsys ysys '5';
	/* Y-axis */
	%line(9, 13, 9, 99, &CX_AXIS., 1, &LINESIZE.); /* Left line of the graph
box */
	%MACRO Y_TL(); /* Ticks and labels */
		%DO I = - &OE_MAX. %TO &OE_MAX. %BY &Y_INTERVAL.;
			%LET HEIGHT = %SYSEVALF(&I. / &OE_MAX. * 86 / 2 + 56); /* Y-
coordinate of the tick */
			%line(8.5, &HEIGHT., 9, &HEIGHT., &CX_AXIS., 1, &LINESIZE.);
/* Tick */
			%label(8, &HEIGHT., "&I.", &CX_AXIS., 0, 0, &FONTSIZE.,
verdana, <); /* Label */
		%END;
	%MEND;
	%Y_TL();
	/* X-axis */
	%line( 9, 56, 99, 56, &CX_AXIS_L.,     1, &LINESIZE.); /* Horizontal line
at the middle of the graph box */
	%line( 9, 13, 99, 13, &CX_AXIS., 1, &LINESIZE.); /* Bottom line of the
graph box */
	%MACRO X_TL(); /* Ticks and labels */
		%LET NTICKS = %SYSFUNC(int(&N_MAX. / &X_INTERVAL.)); /* Number of
ticks */
		%DO I = 0 %TO &NTICKS.;
			%LET LABEL = %SYSEVALF(&I. * &X_INTERVAL.);
			%LET COORD = %SYSEVALF(&LABEL. / &N_MAX. * 90 + 9); /* X-
coordinate of the tick */
			%line(&COORD., 11, &COORD., 13, &CX_AXIS., 1, &LINESIZE.); /*
Tick */
			%label(&COORD., 9, "&LABEL.",  &CX_AXIS., 0, 0, &FONTSIZE.,
verdana, +); /* Label */
		%END;
	%MEND;
	%X_TL();
	/* Closing the graph box */
	%line( 9, 99, 99, 99, &CX_AXIS., 1, &LINESIZE.); /* Top line of the graph
box */
	%line(99, 13, 99, 99, &CX_AXIS., 1, &LINESIZE.); /* Right line of the graph
box */
	/* Axis labels */
	%label(3, 56, "Cumulative Observed minus Expected", &CX_AXIS., 90, 0, 4,
verdana, +);
	%label(54, 3, "Number of procedures", &CX_AXIS., 0, 0, 4, verdana, +);
run;



/* --- O-E curve --- */

/* Calculating the coordinates of each point of the curve, which will be used for
several graphic elements */
data cc;
	set cc;
	x_coord = n / &N_MAX. * 90 + 9;
```

```
        y_coord = oe / &OE_MAX. * 86 / 2 + 56;
run;
/* Writing the annomac table from data */
data curve (keep = hsys xsys ysys color x y function line size);
        format color $8. function $8.;
        retain hsys xsys ysys '5' line 1 size &LINESIZE.;
        if _n_ = 1 then do; /* Zero point */
                function = "MOVE";
                x = 9;
                y = 56;
                output;
        end;
        set cc (drop = x z);
        function = "DRAW";
        x = x_coord;
        y = y_coord;
        /* Color depending on the sequence type */
        if group_type = "seq_x" then color = "&CX_SEQX.";
        if group_type = "seq_z" then color = "&CX_SEQZ.";
        if group_type = "stand" then color = "&CX_STD.";
        output;
run;



/* --- Strips showcasing sequences --- */

/* Writing the annomac table from data */
/* Each strip is a polygon delimited horizontally by the start and the end of a
sequence and vertically by the O-E curve and the bottom of the graph */
/* The polygons start at their {left, bottom} point then end at their {right,
bottom} point */
data strips (keep = hsys xsys ysys color x y function line style);
        format color $8. function $8. style $12.;
        retain hsys xsys ysys '5' line 1 style "msolid" lag_x_coord lag_y_coord;
        set cc (drop = x z);
        /* Strip color depending on the sequence type */
        if group_type = "seq_x" then color = "&CX_SEQX_L.";
        if group_type = "seq_z" then color = "&CX_SEQZ_L.";
        /* First procedure of a sequence */
        if group_type in ("seq_x" "seq_z") and group ne lag(group) then do;
                /* First point : {left, bottom} */
                function = "poly";
                x = lag_x_coord; /* The interval between the first procedure in a
sequence and the procedure before it is considered a part of the sequence */
                y = 13;
                if x = . then x = 9; /* In case the sequence starts at the zero-
point */
                output;
                /* Second point : {left, top} */
                function = "polycont";
                x = lag_x_coord;
                y = lag_y_coord;
                if x = . then do; /* In case the sequence starts at the zero-point
*/
                        x = 9;
                        y = 56;
                end;
                output;
        end;
        /* procedure within a sequence */
        if group_type in ("seq_x" "seq_z") and signal = " " then do;
                /* Points along the O-E curve : {*, top} */
                function = "polycont";
                x = x_coord;
                y = y_coord;
                output;
        end;
```

```
        /* Last procedure of a sequence */
        if signal ne " " then do;
                /* Second-last point : {right, top} */
                function = "polycont";
                x = x_coord;
                y = y_coord;
                output;
                /* Last point : {right, bottom} */
                function = "polycont";
                x = x_coord;
                y = 13;
                output;
        end;
        /* Retaining the coordinates for the next procedure */
        lag_x_coord = x_coord;
        lag_y_coord = y_coord;
run;

/* Adding the white lines that separate adjacent sequences */
data wlines (keep = hsys xsys ysys color x y function line style size);
        format color $8. function $8. style $12.;
        retain hsys xsys ysys '5' line 1 style "msolid" lag_x_coord 2;
        set cc (drop = x z);
        /* We draw a line if the last procedure and the current one belong in two
different sequences */
        if group_type in ("seq_x" "seq_z") and lag(group_type) in ("seq_x" "seq_z")
and group ne lag(group) then do;
                /* Start of the line */
                function = "MOVE";
                size = &LINESIZE. * 2;
                x = (x_coord + lag_x_coord) / 2;
                y = 99;
                color = "white";
                output;
                /* End of the line */
                function = "DRAW";
                size = &LINESIZE. * 2;
                x = (x_coord + lag_x_coord) / 2;
                y = 13;
                color = "white";
                output;
        end;
        /* Retaining the coordinates for the next procedure */
        lag_x_coord = x_coord;
run;



/* --- Circles displaying the O-E within sequences --- */

/* Calculating the coordinates of the circles */
data cc;
        set cc;
        /* Coordinates are stored on the last sequence of each procedure */
        if signal ne " " then do;
                coord_x_sig = (n + n_seq_start) / 2 / &N_MAX. * 90 + 9; /* Middle of
the sequence */
                coord_y_sig = 21;
        end;
run;

/* Circles : writing the annomac table from data */
data circles (keep = hsys xsys ysys color x y function line style size angle
rotate);
        format color $8. function $8. style $12.;
        retain hsys xsys ysys '5' line 1 style "psolid" function "pie" angle 360
rotate 360;
        set cc (drop = x z where = (coord_y_sig ne .));
```

```
        x = coord_x_sig;
        y = coord_y_sig;
        /* Outline of the circles (actually bigger circles) */
        if signal = "x" then color = "&CX_SEQX_L.";
        if signal = "z" then color = "&CX_SEQZ_L.";
        size = 5;
        output;
        /* Inside of the circles (white, smaller circles) */
        color = "white";
        size = 4;
        output;
run;

/* O-E values : writing the annomac table from data */
data oe_values (keep = hsys xsys ysys color x y function style size angle rotate
text position);
        format color $8. function $8. style $12. text $39.;
        retain hsys xsys ysys '5' line 1 style "verdana" function "label" size 4
angle 0 rotate 0 position "+";
        set cc (drop = x z where = (coord_y_sig ne .));
        x = coord_x_sig;
        y = coord_y_sig;
        if group_type = "seq_x" then color = "&CX_SEQX.";
        if group_type = "seq_z" then color = "&CX_SEQZ.";
        text = compress(put(group_oe, signe.));
run;



/* --- Final annomac table --- */

/* Concatenating annomac tables, background to foreground */
data graph;
        set strips
                wlines
                circles
                oe_values
                axis
                curve;
run;





/* ---------------------------------------- */
/* ---------- Graph file output ---------- */
/* ---------------------------------------- */



filename pngname "&PNG_PATH.";
goptions device = png xpixels = &XPX. ypixels = &YPX. gsfmode = replace gsfname =
pngname;
proc ganno annotate = graph;
run;
quit;
```

Supplemental File 4: Patient's surgical outcome and preoperative risk score development

Surgical outcome was a composite morbidity-mortality assessment criterion including, in the operating room during the initial surgery or within 30 days following the initial surgery, the detection of at least one of the following major adverse events during the period of hospitalization when the procedure was performed or during a subsequent hospitalization:

- Severe complication:
  - Intraoperative complication that occurred during the initial surgery between the time of entry and exit from the operating room: multi-organ failure, shock (hemorrhagic, cardiogenic, septic, or anaphylactic), cardiac arrest/ myocardial infarction, major hemorrhage, hemodynamic instability, accidental wound, material oversight, procedure error, or surgical conversion;
  - Postoperative complication that occurred after discharge from the operating room on the same day or within 30 days following the initial surgery: general (multi-organ failure, implantable medical device or graft failure), infectious (sepsis, organ or surgical site infection, deep infection, implantable medical device or graft infection, pulmonary infection), hemorrhagic (hemorrhage, hemorrhagic shock, deep hematoma, hemarthrosis), parietal (eventration or evisceration), cardiopulmonary (cardiac arrest, myocardial infarction, acute heart failure, acute respiratory failure), neurological (coma, stroke), abdominopelvic (anastomotic complication, fistula, ascites, acute liver failure, intestinal obstruction, digestive necrosis, acute renal failure, pyeloureteral obstruction), orthopedic (fracture, dislocation, ischemia, compartment syndrome, paralysis), cervical (recurrent paralysis, hypoparathyroidism, suffocating hematoma), functional (undernourishment, transit stop);
- Unplanned reoperation for complications related to the initial surgery, including any return of the patient to the operating room for a new surgical procedure with open, video, endoscopic, or interventional radiology approach;
- Postoperative transfer in intensive or intermediate care unit related to organ failure during or after the initial surgery with extended stay (of at least two nights in intensive care unit, at least five nights in intermediate care unit) or ended by death;
- Intraoperative/postoperative death, regardless of the cause.

A patient preoperative risk score was specifically developed using an independent train dataset (a 50% random sample) of 3,644 operations performed by the same cohort of surgeons at a different period, from January 1st, 2022, to October 31st, 2022. The following variables were systematically considered for inclusion in the models:

- Surgical procedure (767 distinct types of procedures spanning various surgical specialties)
- Surgical indication of the operation based on the chapter of the ICD-10 codes
- Scheduling of the operation (urgent, semi-urgent, elective)
- Type of anesthesia (general, regional, local) and surgical approach (open, videoscopic, endoscopic, robot)
- Patient demographics (age, sex) and socio-economic status (individual precarious situation and median income of the municipality of residence in quartiles)
- ASA physical status classification system (from 1 to 5)
- Comorbidities including critical condition, current pregnancy, obesity BMI $\geq$ 30 kg/m², malnourishment, tobacco/alcohol or other drug addictions, open wound, surgical site infection, sepsis, endocarditis, cancer, neoadjuvant treatment, immune deficiency,

coagulopathy, anticoagulant or antiaggregation treatment, blood transfusion, coma, limb paralysis, other neurological disorder, confusion, dementia, depression, cardiovascular disease, neurovascular disease, peripheral arterial disease, cardiac arrhythmia, chronic heart failure, hypertension, diabetes, dyslipidemia, pulmonary artery systolic pressure > 60 mmHg, chronic renal failure, acute renal failure, chronic respiratory failure, chronic obstructive pulmonary disease, liver disease, rheumatic pathology, and hypoparathyroidism.

Considered all those potential confounders, models were subsequently trained using the operations from the independent dataset, grouping certain specialties due to low event rates, as follows: cardiac with thoracic surgery, digestive with endocrine surgery, and orthopedic with urologic and gynecologic surgery. Variables were systematically selected using an automated stepwise logistic regression approach (entry threshold p = 0.20, exit threshold p = 0.10), enabling us to retain a specific set of variables for each specialties group. The obtained beta coefficients were then applied to the operations of the present observation study period (a 100% exhaustive sample of procedures performed by the cohort of surgeons, from November 1, 2020, to December 31, 2021), allowing us to estimate the probability of adverse event occurrence for each operation.

Models prediction performance was evaluated based on their calibration and discrimination with C-statistics and corresponding AUC on the implementation train dataset and on the observation test dataset for accuracy (external validation).

Accuracy	Calibration	Discrimination
Train : 86.5%<br>Test : 82.2%	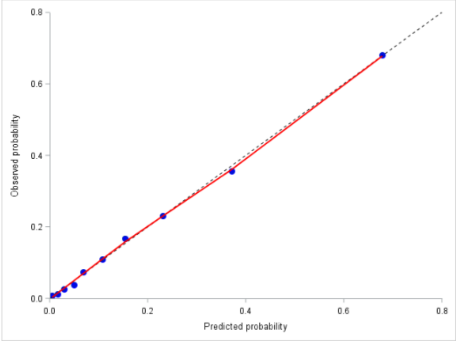	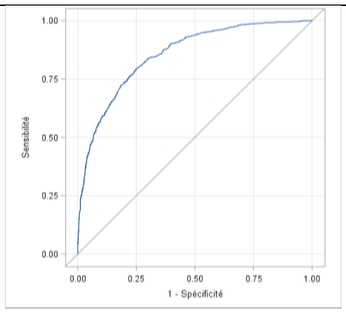<br>C-Stat:<br>0.854 (0.838-0.870)

The choice of variables used for the adjustment was tailored to the specific needs of our study. However, it is possible – and even worthwhile – to include other variables, particularly proxies for the surgeon's experience. It is also feasible to use hierarchical models (such as mixed models, GEE, etc.) instead of standard logistic models to better capture the performance level specific to each surgeon (and potentially account for case-mix variations not detected through adjustment). However, this would require having prior data for each surgeon, making it impossible to include new surgeons along the way, thereby limiting the tool's reproducibility.

Supplemental Figure 1: Flowchart

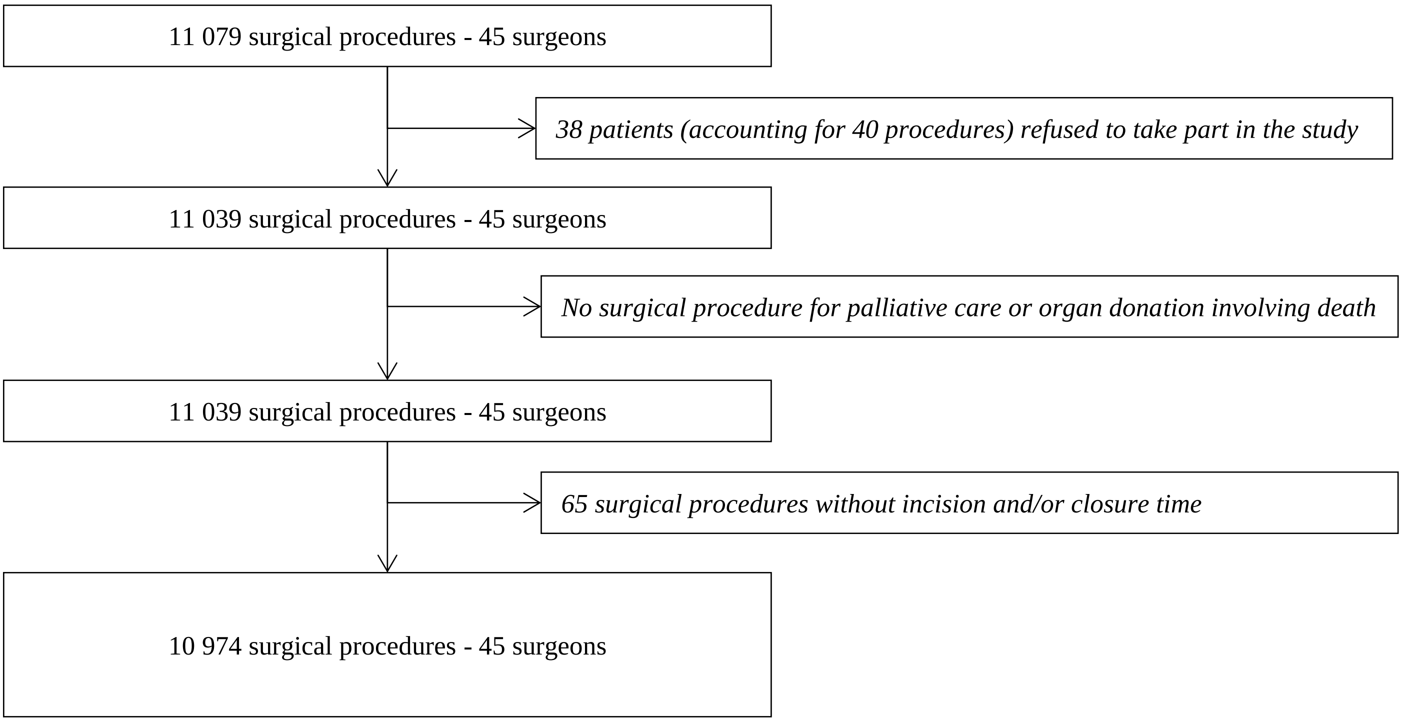

Supplemental figure 2: Risk-adjusted O-E CUSUM chart for a single digestive surgeon – Unplanned reoperations

A. Observed minus Expected (O-E) chart

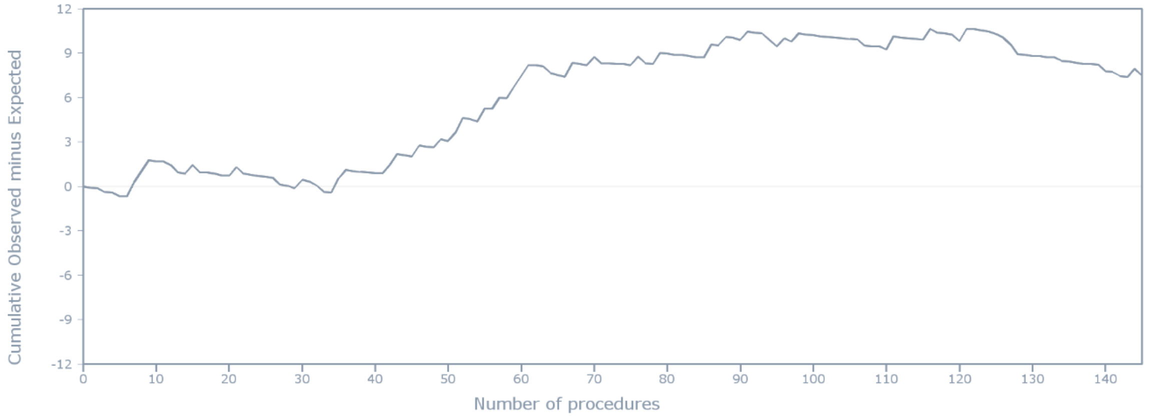

B. CUmulative SUM (CUSUM) chart

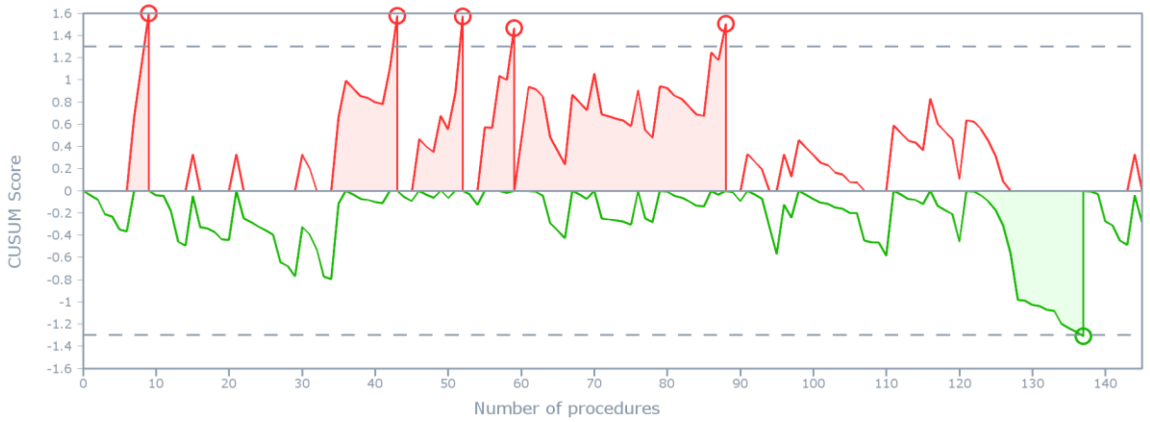

C. Observed minus Expected CUmulative SUM (O-E CUSUM) chart

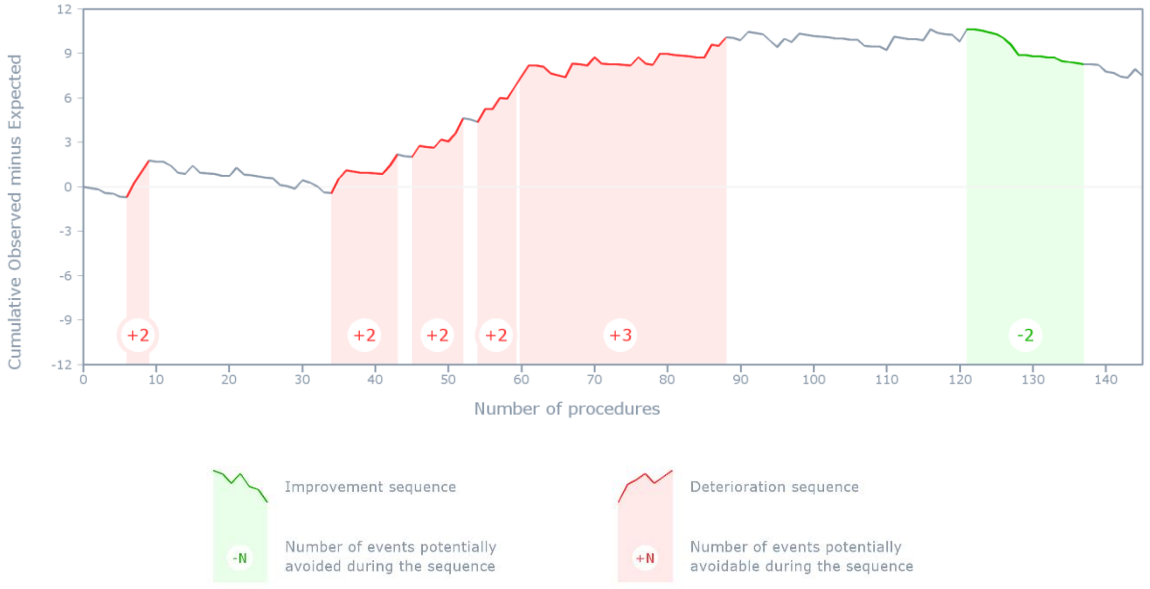

Five deteriorations sequences are observed (procedures 6-9, 35-43, 46-52, 55-59 and 60-88), followed by a plateau and then a sequence of improvement (122-137). Overall, the difference between the observed and expected events was +7.5, indicating 7.5 potentially avoidable events during this period.

Supplemental Figure 3: O-E CUSUM charts for the 45 surgeons

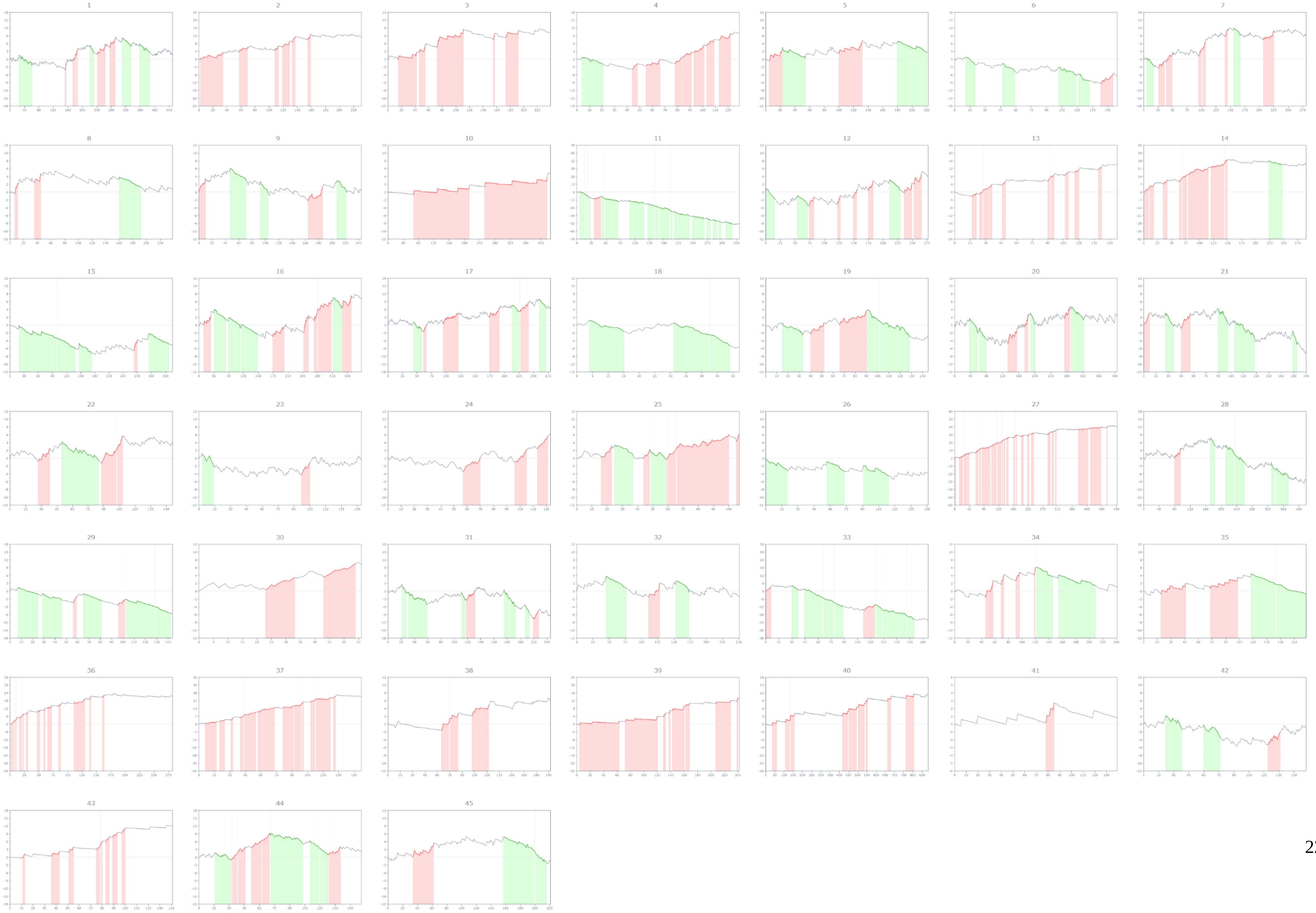

Supplemental figure 4: Comparison with Sherlaw-Johnson's chart

Sherlaw-Johnson proposed a risk-adjusted O-E chart with control bands based on a two-sided CUSUM chart. Afin de mieux identifier les différences entre nos outils respectifs, nous les avons comparé avec les données du chirurgien digestif de notre exemple (see figure 3).

Sherlaw-Johnson C. A method for detecting runs of good and bad clinical outcomes on Variable Life-Adjusted Display (VLAD) charts. Health Care Manag Sci. 2005 Feb;8(1):61-5. doi: 10.1007/s10729-005-5217-2. PMID: 15782513.

A. Sherlaw-Johnson's Risk Adjusted Observed minus Expected (RA O-E) chart with CUSUM based control limits

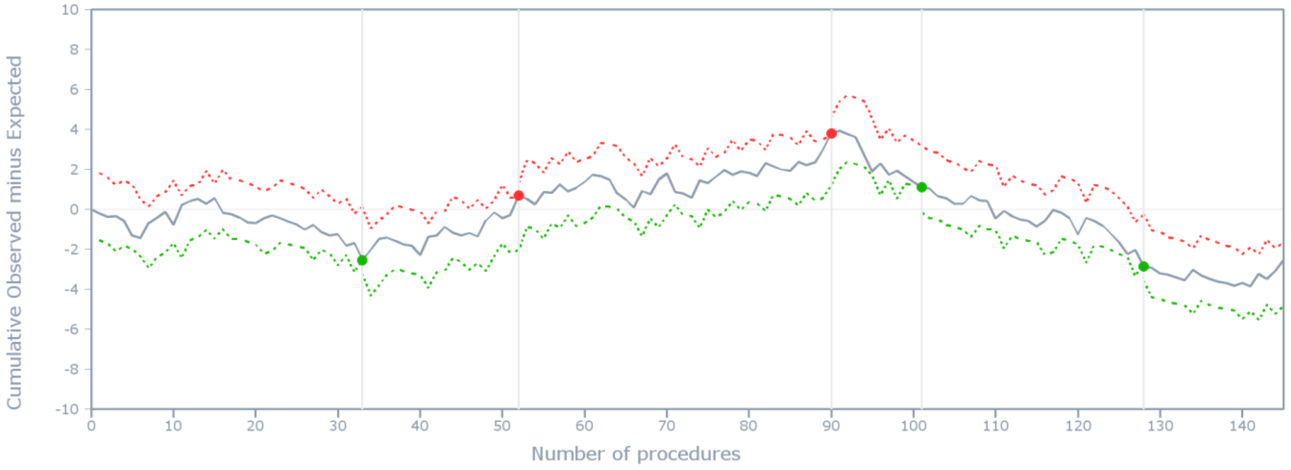

B. Proposed Risk-Adjusted Observed minus Expected CUmulative SUM (RA O-E CUSUM) chart

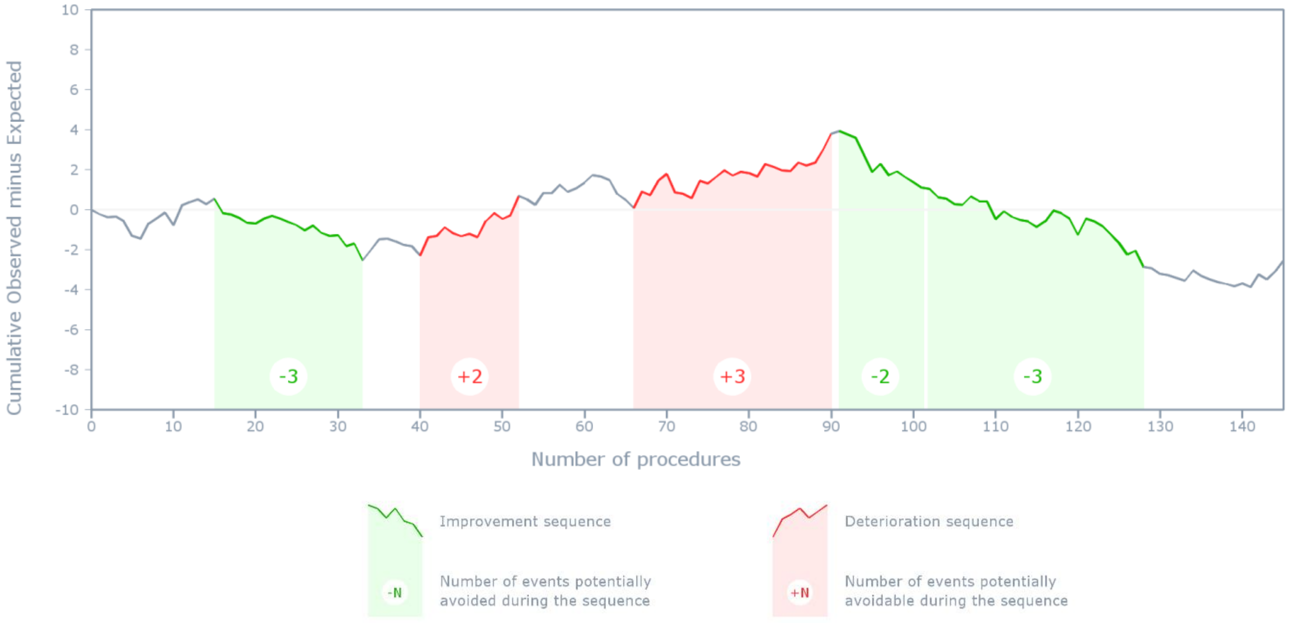

The red and green dashed lines on Sherlaw-Johnson's chart represent the upper and lower control limits, respectively. They are based on the CUSUM chart and correspond to the theoretical value that the O-E curve should reach for the CUSUM to detect a signal. When the O-E curve reaches the control limit, the CUSUM signals, and a change in surgical performance is detected (a red point for deterioration or a green point for improvement). The underlying CUSUM chart is then reset, and the control limits are recalculated to account for the fact that the process no longer starts from zero but from where the signal was detected. Each reset is marked by a vertical gray line.

Sherlaw-Johnson's chart has the clear advantage of directly applying control limits to the O-E chart, which have the statistical power of the CUSUM. It highlights the evolution of the O-E curve and how it approaches the control limits. However, it overlooks the sequence of changes in surgical performance and may potentially lead the surgeon to focus only on the most recent procedures before a signal was detected. For example, a signal for deterioration was detected at procedure 52: the chart might prompt the surgeon to only investigate procedures 50-52, even though surgical performance was statistically deteriorated since procedure 41. The same applies to the improvement sequence detected at procedure 128, which might lead the surgeon to focus on procedures 125-128, while performance was actually statistically improved since procedure 102 (see CUSUM chart).

Instead of overlaying control limits (as with the preceding approach), we directly displayed the sequences of performance deterioration and improvement. This draws the user's attention to the entire statistically significant sequence rather than just the most recent procedures that triggered the signal (see the Sherlaw-Johnson chart), while also allowing for the chronological identification of when the observed change began. To use an everyday comparison, we want to encourage surgeons to investigate the entire process that filled the cup (with statistical significance) and when it did begin, not just the final drops that caused it to overflow (not necessarily statistically significant). The proposed tool handles multiple signals, even when they are close together, and is relatively simple and straightforward to interpret (compared to the V-mask). It may also be easier for surgeons without a statistical background to use, as they do not need to understand the properties of the CUSUM or the concept of control limits. Finally, the magnitude of the sequences is quantified, giving the surgeon a concrete view of the impact of performance changes in terms of the number of events.
